# Effects of severe anthropogenic disturbance on the heart rate and body temperature in free-living greylag geese (*Anser anser*)

**DOI:** 10.1093/conphys/coac050

**Published:** 2022-07-25

**Authors:** Claudia A F Wascher, Walter Arnold, Kurt Kotrschal

**Affiliations:** Behavioural Ecology Research Group, School of Life Sciences, Anglia Ruskin University, Cambridge, CB1 1PT UK; Research Institute of Wildlife Ecology, University of Veterinary Medicine, A-1160 Vienna, Austria; Core Facility Konrad Lorenz Forschungsstelle for Behaviour and Cognition, University of Vienna, 4645 Grünau im Almtal, Austria; Department of Behavioural Biology, University of Vienna, 1090 Vienna, Austria

**Keywords:** wildlife conservation, heart rate, greylag geese, emotional arousal, anthropogenic disturbance, animal welfare

## Abstract

Anthropogenic disturbances are a major concern for the welfare and conservation of wildlife. We recorded heart rate and body temperature of 20 free-living greylag geese in response to a major regularly re-occurring anthropogenic disturbance—New Year’s Eve fireworks. Heart rate and body temperature were significantly higher in the first and second hour of the new year, compared with the same hour on the 31st of December, the average during December and the average during January. Heart rate and body temperature was not significantly affected by sex or age. From 0200 to 0300 onwards, 1st of January heart rates did not significantly differ from the other periods; however, body temperatures were significantly increased until 0300–0400. From 0400 to 0500, heart rate was not affected by any of the investigated factors, whereas body temperature was significantly increased on the 1st of January compared with the 31st of December and the December average but not compared with the January average. To conclude, our results show that New Year’s Eve fireworks cause a substantial physiological response, indicative of a stress response in greylag geese, which is costly in terms of energy expenditure.

## Teaser

New Year's fireworks present a major, regularly occurring disturbance, which can significantly affect wildlife and be a concern for the welfare and conservation. We recorded heart rate and body temperature of 20 free-living greylag geese. Heart rate and body temperature were significantly increased in response to the fireworks. Our results show that New Year’s Eve fireworks to cause a substantial stress response in greylag geese.

## 1. Introduction

Anthropogenic disturbances, such as noise or light pollution, human presence or motor vehicles, are increasingly becoming a major concern for the welfare and conservation of wildlife (Marion *et al.,* 2020; Corradini *et al.,* 2021; Jerem and Mathews, 2021). Disturbances can result in short-term to long-term changes in the behaviour and physiology of individuals (Bejder *et al.,* 2006; New *et al.,* 2014). Globally, species increase their nocturnality in response to human disturbance (Gaynor *et al.,* 2018). Physiological activation, for example, via the sympathetic branch of the automatic nervous system, causing an increase in heart rate and body temperature (Bartholomew *et al.,* 1964; Cabanac and Guillemette, 2001; Carere and Vanoers, 2004), which helps organisms to cope with environmental challenges [e.g. temperature stress (Bartholomew *et al.,* 1964, Al-Haidary, 2004, Alam *et al.,* 2011), hunger (Mesteig *et al.,* 2000; De Jong *et al.,* 2002; Savory and Kostal, 2006), agonistic encounters (Wascher, 2021), predator exposure (Oulton *et al.,* 2013)] and maintain homeostasis (von Holst, 1998). However, this comes at the cost of increased energy expenditure (Weimerskirch *et al.,* 2002; Wascher *et al.,* 2018; Halsey *et al.,* 2019). This is costly, as animals are limited in their actions by their energy throughput, the amount of energy they consume and use (McNab, 2022). Increases in heart rate and body temperature can also be an indication of emotional arousal and poor welfare (von Borell *et al.,* 2007; Wascher, 2021). Chronic anthropogenic noise has been shown to decrease baseline corticosterone in birds and, conversely, increasing acute stressor-induced corticosterone (Kleist *et al.,* 2018). Bird nestlings in noisy areas had shorter telomere lengths (Grunst *et al.,* 2021). Via such mechanisms, anthropogenic disturbances can negativity affect individuals’ fitness (Daan *et al.,* 1996; Fowler, 1999), for example, decrease hatching success or body condition (Kleist *et al.,* 2018). Combined effects of anthropogenic noise and artificial light affect activity patterns in birds (Dominoni *et al.,* 2020), community structure (Willems *et al.,* 2022) and chick development (Ferraro *et al.,* 2020).

Biologging technologies to measure heart rate can be used to assess effects of anthropogenic disturbance on wildlife. This is especially relevant, as studies have shown that individuals can show pronounced activation of the physiological stress response in the absence of obvious behavioural changes. For example, American black bears, *Ursus americanus*, and bighorn sheep, *Ovis canadensis*, showed no behavioural responses but significantly increased heart rate in response to anthropogenic disturbances, such as drone overflights and vehicle traffic (MacArthur *et al.,* 1979; Ditmer *et al.,* 2015).

Anthropogenic noise causes elevated heart rates in farm animals (Talling *et al.,* 1996), fish (Graham and Cooke, 2008) and marine mammals (Hinde *et al.,* 2018). Additionally, in response to a large-scale military manoeuvre moose, *Alces alces*, showed behavioural changes, i.e*.* decrease in flush distance, and physiological changes, i.e. higher maximum heart rates (Andersen *et al.,* 1996). Direct human contact can activate the physiological stress response in wild animals; for example, Brown bears, *Ursus arctos,* significantly increased heart rate in response to dog hunts and human encounters (Le Grand *et al.,* 2019). Human approach towards nesting birds may not always lead to females leaving the nest, but activation of the physiological stress response can increase energy expenditure (Magellanic penguins, *Spheniscus magellanicus*: Fowler, 1999; Yellow-eyed penguin, *Megadyptes antipodes:*Ellenberg *et al.,* 2013; wandering albatrosses, *Diomedea exulans*: Weimerskirch *et al.,* 2002). In contrast, other populations were described to be more resilient; for example, nesting American oystercatchers, *Haematopus palliates*, did not significantly increase heart rate in response to a variety of human disturbances, including human approach, off-road vehicles and aircraft overflights (Borneman *et al.,* 2014), and greylag geese did not significantly increase heart rate in response to familiar humans approaching (Wascher *et al.,* 2011). Whether or not a heart rate increase can be measured upon such events may be a matter of previous habituation.

A major regularly re-occurring world-wide anthropogenic disturbance are New Year’s Eve firework celebrations, causing noise and light pollution and major anxiety in pet animals. Hence, it is a significant welfare concern (dogs, *Canis familiaris*: Levine and Mills, 2008, Dale *et al.,* 2010, Gates *et al.,* 2019, Gähwiler *et al.,* 2020, Riemer, 2020; horses, *Equus caballus*: Gronqvist *et al.,* 2016). A study on several species of captive zoo animals showed no changes in behaviour in response to fireworks in most species (Rodewald *et al.,* 2014). However, birds have been shown to take flight shortly after midnight and move in the air for at least 45 minutes in response to fireworks on New Year’s Eve in the Netherlands (Shamoun-Baranes *et al.,* 2011). Besides these few studies, the effects of fireworks on wildlife are largely unknown.

In the present study, we investigate heart rate and body temperature responses of free-flying greylag geese in response to New Year’s Eve celebrations. We expected a significant increase in both, heart rate and body temperature, in response to the fireworks. Additionally, we investigated any effects of sex and age. Previously, heart rate differences between the sexes have been described only during the reproductive season (Wascher *et al.,* 2018) and specific behavioural contexts (Wascher *et al.,* 2012). Hence, in this study we do not expect different responses between males and females. In respect to age, we were interested whether older individuals, who have experienced multiple New Year’s Eve celebrations, responded less strongly, as they might have habituated to fireworks previously or alternatively sensitized, meaning that response to fireworks would increase over time (Riemer, 2020). Quantifying the impact of fireworks onto the short-term physiological stress response can help to understand the impact of anthropogenic disturbances for wild animals.

## 2. Material and Methods

### 2.1. Study site

The present study was conducted in a non-migratory free-living flock of greylag geese in the Almtal, Upper Austria. At the time of data collection, the flock consisted of approximately 150 individuals, marked with coloured leg bands for individual identification. The geese are unrestrained and freely roam the valley from the lake Almsee, on which they usually roost at night (47.747793°, 13.956805°) to the Konrad Lorenz research station (47.814143°, 13.948519°). At the research station, the flock is supplemented with pellets and grain twice daily at 0800 and 1500 hours during the winter months and at 1700 hours during the summer months. Both hand-raised and goose-raised flock members are habituated to the close presence of humans and they neither show avoidance if approached up to a distance of 1 m nor excrete elevated levels of corticosterone metabolites following such situations (Scheiber *et al.,* 2005) or significantly change heart rate when familiar humans approach (Wascher *et al.,* 2011). New Year’s Eve celebrations and fireworks are held in several nearby villages, including Grünau im Almtal and directly at the Almsee, where the geese roost at night. Geese are subjected to visual as well as auditory stimuli from the fireworks. Most of the firework activity starts at 0000 on the 1st of January and lasts for several minutes. We did not collect behavioural data from the focal individuals in this study; however, from anecdotal observations we know that geese take flight during the fireworks and circle over the Almsee but remain on the roosting site.

### 2.2. Data collection and analysis

A total of 25 individuals (8 females/17 males) were fitted fully implanted transmitter packages (60 × 30 × 11 mm, ~60 g; for further technical details and implantation procedure, see Wascher and Kotrschal, 2013, Wascher *et al.,* 2018). Both heart rate and body core temperature were stored as 2-minute means in the implant over its lifetime and downloaded after electronic packages were surgically removed. Data were recorded during 2005 New Year’s Eve from 20 individuals (6 females/14 males). One male individual was sampled twice during 2005 and 2006. Age of focal individuals ranged from 1 to 12 years (average ± standard deviation = 4.714 ± 2.813). Raw data were filtered with a moving average to remove biologically implausible outlier values. We calculated mean values per hour of the day. We compared daily heart rate pattern on the 31st of December compared with the 1st of January, average values for the month of December and average values for the month of January. Additionally, we calculated mean value over 1 hour (e.g. 00–01, 01–02, 02–03) on the 31st of December and 1st of January and average over the entire month of December and January. Data were analysed using R version 4.0.3 (The R Foundation for Statistical Computing, Vienna, Austria; http://www.r-project.org). We calculated general linear mixed model using the function lme in the package nlme (Pinheiro *et al.,* 2020) with Gaussian error distribution and mean heart rate as well as body temperature as response variable. We calculated separate models for the hours 0000–0100, 0100–0200, 0200–0300, 0300–0400, 0400–0500 and 0500–0600, until no significant difference between different periods could be detected anymore. Period (31st December, 1st January, average December, average January), sex and age were included as explanatory variables. Various model diagnostics were employed to confirm model validity (visual inspection of distribution of residuals, qq plots, residuals plotted against fitted values), none of which suggested violation of model assumptions. To assess multicollinearity between fixed factors, we calculated variance inflation factors (VIFs) using the vif function in the package car (Fox and Weisberg, 2011). VIFs for all factors were below 1.5, indicating that there was no issue with multicollinearity (Zuur *et al.,* 2009). For each model, we fitted individual identity as a random term to control for the potential dependence associated with multiple samples from the same individuals. To describe the variance explained by our models, we provide marginal and conditional *R2* values that range from 0 to 1 and describe the proportion of variance explained by the fixed and random effects combined, respectively (Nakagawa and Schielzeth, 2013). We calculated marginal and conditional *R2* values using the r.squaredGLMM function in MuMIn (Bartoń, 2019).

**Table 1:** Results of the general linear model investigating factors affecting heart rate (**A**) and body temperature (**B**) between 0000–0100, 0100–0200, 0200–0300, 0300–0400, 0400–0500 and 0500–0600

	Parameters	Estimate ± SE	*df*	*t*-value	*P*
0000–0100					
(A) Heart rate	(Intercept)	−3477.243 ± 2379.94	61	−1.461	0.149
*R2* marginal: 55%, *R2* conditional: 9%	**Period (1 Jan relative to 31 Dec)**	**−55.424 ± 6.465**	**61**	**−8.571**	**<0.001**
	**Period (1 Jan relative to Dec average)**	**−60.569 ± 6.465**	**61**	**−9.367**	**<0.001**
	**Period (1 Jan relative to Jan average)**	**−55.721 ± 6.46**	**61**	**−8.617**	**<0.001**
	Sex	−8.933 ± 7.171	17	−1.245	0.229
	Age	1.803 ± 1.19	17	1.514	0.148
(B) Body temperature	(Intercept)	70.924 ± 161.338	61	0.439	0.661
*R2* marginal: 24%, *R2* conditional: 64%	**Period (1 Jan relative to 31 Dec)**	**−1.242 ± 0.13**	**61**	**−9.505**	**<0.001**
	**Period (1 Jan relative to Dec average)**	**−1.284 ± 0.13**	**61**	**−9.822**	**<0.001**
	**Period (1 Jan relative to Jan average)**	**−1.18 ± 0.13**	**61**	**−9.026**	**<0.001**
	Sex	−0.427 ± 0.479	17	−0.892	0.384
	Age	−0.015 ± 0.08	17	−0.194	0.848
0100–0200					
(A) Heart rate	(Intercept)	−2568.699 ± 1903.819	59	−1.349	0.182
*R2* marginal: 21%, *R2* conditional: 22%	**Period (1 Jan relative to 31 Dec)**	**−0.973 ± 0.125**	**59**	**−7.728**	**<0.001**
	**Period (1 Jan relative to Dec average)**	**−1.074 ± 0.125**	**59**	**−8.533**	**<0.001**
	**Period (1 Jan relative to Jan average)**	**−0.989 ± 0.126**	**59**	**−7.805**	**<0.001**
	Sex	−0.328 ± 0.467	17	−0.7	0.492
	Age	−0.016 ± 0.078	17	−0.212	0.834
(B) Body temperature	(Intercept)	72.663 ± 157.464	59	0.461	0.646
*R2* marginal: 20%, *R2* conditional: 66%	**Period (1 Jan relative to 31. Dec)**	**−0.973 ± 0.124**	**59.144**	**−7.83**	**<0.001**
	**Period (1 Jan relative to Dec average)**	**−1.074 ± 0.124**	**59.144**	**−8.645**	**<0.001**
	**Period (1 Jan relative to Jan average)**	**−0.989 ± 0.125**	**59.036**	**−7.908**	**<0.001**
	Sex	−0.328 ± 0.488	17.04	−0.671	0.51
	Age	−0.016 ± 0.082	16.964	−0.203	0.841
0200–0300					
(A) Heart rate	(Intercept)	−2330.824 ± 1529.329	59	−1.524	0.132
*R2* marginal: 11%, *R2* conditional: 36%	Period (1 Jan relative to 31 Dec)	−2.872 ± 2.91	59	−0.987	0.327
	Period (1 Jan relative to Dec average)	−2.923 ± 2.91	59	−1.004	0.319
	Period (1 Jan relative to Jan average)	2.598 ± 2.934	59	0.885	0.379
	Sex	−3.294 ± 4.573	17	−0.72	0.481
	Age	1.199 ± 0.765	17	1.567	0.135
(B) Body temperature	(Intercept)	52.334 ± 153.373	59	0.341	0.734
*R2* marginal: 8%, *R2* conditional: 80%	**Period (1 Jan relative to 31 Dec)**	**−0.496 ± 0.108**	**59**	**−4.592**	**<0.001**
	**Period (1 Jan relative to Dec average)**	**−0.572 ± 0.108**	**59**	**−5.299**	**<0.001**
	**Period (1 Jan relative to Jan average)**	**−0.48 ± 0.108**	**59**	**−4.42**	**<0.001**
	Sex	−0.255 ± 0.455	17	−0.561	0.582
	Age	−0.006 ± 0.076	17	−0.089	0.929
0300–0400					
(A) Heart rate	(Intercept)	−2688.979 ± 1630.469	59	−1.649	0.104
*R2* marginal: 8%, *R2* conditional: 35%	Period (1 Jan relative to 31 Dec)	0.545 ± 3.178	59	0.171	0.864
	Period (1 Jan relative to Dec average)	0.033 ± 3.178	59	0.01	0.991
	Period (1 Jan relative to Jan average)	2.323 ± 3.205	59	0.724	0.471
	Sex	−2.869 ± 4.878	17	−0.588	0.564
	Age	1.377 ± 0.815	17	1.688	0.109
(B) Body temperature	(Intercept)	47.319 ± 150.273	59	0.314	0.754
*R2* marginal: 4%, *R2* conditional: 83%	**Period (1 Jan relative to 31 Dec)**	**−0.399 ± 0.104**	**59**	**−3.811**	**<0.001**
	**Period (1 Jan relative to Dec average)**	**−0.382 ± 0.104**	**59**	**−3.65**	**<0.001**
	**Period (1 Jan relative to Jan average)**	**−0.271 ± 0.105**	**59**	**−2.576**	**0.012**
	Sex	−0.189 ± 0.446	17	−0.424	0.676
	Age	−0.004 ± 0.075	17	−0.059	0.952
0400–0500					
(A) Heart rate	(Intercept)	−2235.595 ± 1617.503	59	−1.386	0.171
*R2* marginal: 7%, *R2* conditional: 26%	Period (1 Jan relative to 31 Dec)	−0.453 ± 3.703	59	−0.122	0.903
	Period (1 Jan relative to Dec average)	2.139 ± 3.703	59	0.577	0.565
	Period (1 Jan relative to Jan average)	2.132 ± 3.738	59	0.57	0.57
	Sex	−5.934 ± 4.855	17	−1.222	0.238
	Age	1.154 ± 0.809	17	1.427	0.171
(B) Body temperature	(Intercept)	51.501 ± 147.849	59	0.348	0.728
*R2* marginal: 2%, *R2* conditional: 81%	**Period (1 Jan relative to 31 Dec)**	**−0.295 ± 0.118**	**59**	**−2.491**	**0.015**
	**Period (1 Jan relative to Dec average)**	**−0.285 ± 0.118**	**59**	**−2.407**	**0.019**
	Period (1 Jan relative to Jan average)	−0.185 ± 0.119	59	−1.551	0.126
	Sex	−0.097 ± 0.439	17	−0.22	0.827
	Age	−0.006 ± 0.073	17	−0.09	0.928
0500–0600					
(A) Heart rate	(Intercept)	−1558.818 ± 1747.148	59	−0.892	0.375
*R2* marginal: 36%, *R2* conditional: 38%	Period (1 Jan relative to 31 Dec)	0.378 ± 3.336	59	0.113	0.91
	Period (1 Jan relative to Dec average)	0.08 ± 0.116	59	0.687	0.494
	Period (1 Jan relative to Jan average)	1.237 ± 3.336	59	0.37	0.712
	Sex	−4.315 ± 5.225	17	−0.825	0.42
	Age	0.814 ± 0.42	17	0.932	0.364
(B) Body temperature	(Intercept)	74.265 ± 149.291	59	0.497	0.62
*R2* marginal: 15%, *R2* conditional: 83%	Period (1 Jan relative to 31 Dec)	0.222 ± 0.116	59	1.906	0.061
	Period (1 Jan relative to Dec average)	0.08 ± 0.116	59	0.687	0.494
	Period (1 Jan relative to Jan average)	−0.02 ± 0.114	59	−0.176	0.86
	Sex	−0.016 ± 0.443	17	−0.038	0.97
	Age	−0.018 ± 0.074	17	−0.244	0.81

Significant factors are highlighted in bold. For each model, *R2* marginal value, describing the proportion of variance explained by the fixed and *R2* conditional value describing proportion of variance explained by the random effect (individual).

**Figure 1:**
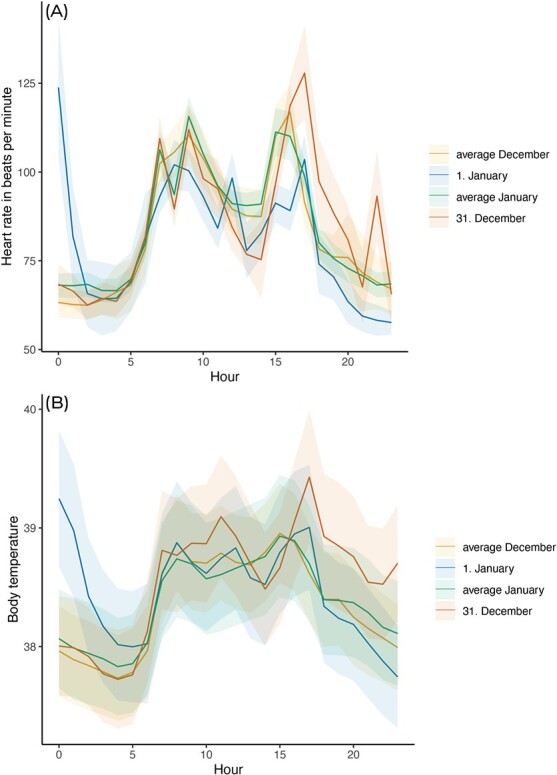
Daily course of (**A**) heart rates and (**B**) body temperature. Solid lines are means, shaded areas indicate standard error between individuals.

**Figure 2:**
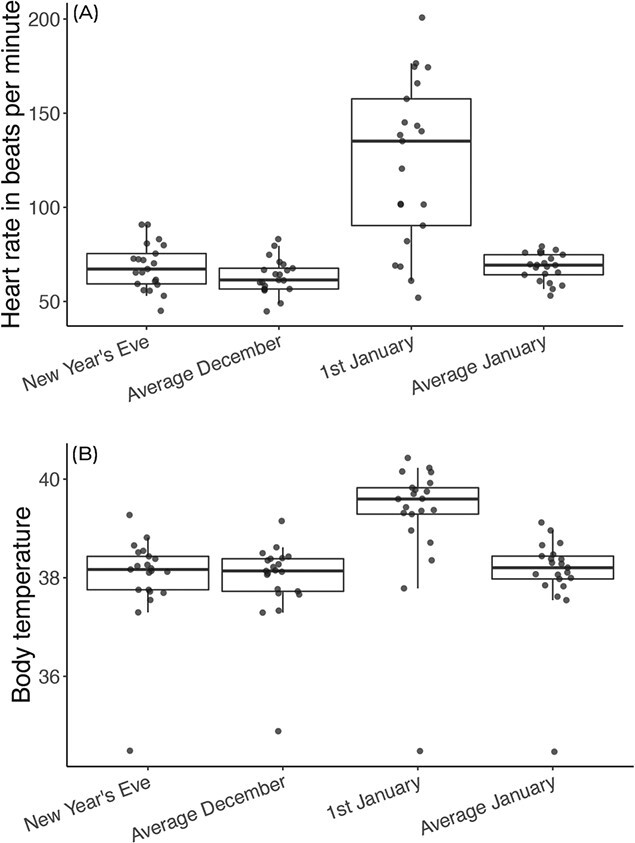
Mean heart rate (**A**) and body temperature (**B**) between 0000 and 0100. Box plots show the median and the interquartile range from the 25th to the 75th percentiles. Whiskers show the bottom 5% to the upper 95% confidence interval. Each data point is an individual’s mean value.

### 2.3. Results

Between 0000 and 0100, heart rate and body temperature were significantly higher in the first hour of the new year, compared with the same hour on the 31st of December, the average during December and the average during January (Table 1; Figs 1 and 2). Compared with average values during the month of December, heart rate increased by 96% and body temperature increased by 3% in the first hour of the new year. Heart rate and body temperature was not significantly associated with sex and age (Table 1). A similar pattern arose between 0100 and 0200, with heart rate and body temperature being significantly higher on the 1st of January compared with the other phases, but sex and age not having an effect (Table 1), reflecting a 31% increase in heart rate and 3% increase in body temperature compared with average December values. Between 0200–0300 and 0300–0400, heart rate was not significantly associated with any factors; however, body temperature was still significantly higher on the 1st of January compared with other phases. Between 0400 and 0500, body temperature was significantly higher on the 1st of January compared with 31st of December and average December values but not average January values. None of the investigated factors significantly affected body temperature between 0500 and 0600 (Table 1).

## 3. Discussion

In the present study we describe a significant increase in heart rate and body temperature in response to a major regularly re-occurring anthropogenic disturbance, New Year’s Eve firework celebrations. Heart rate was significantly increased from 0000 to 0200 compared with control periods. Similarly, also body temperature increased in response to the disturbance and only returned to baseline between 0500 and 0600 on the 1st of January. Although fireworks are well described as a major stressor in pets and domestic animals (Levine and Mills, 2008; Dale *et al.,* 2010; Gronqvist *et al.,* 2016; Gates *et al.,* 2019; Gähwiler *et al.,* 2020), effects on wildlife remain largely unexplored (Shamoun-Baranes *et al.,* 2011). We cannot conclusively tell whether the implanted geese were responding to the noise pollution or light pollution caused by the fireworks or to a combination of both. It has to be noted that the geese in our study were generally habituated to human presence and, for example, did not significantly increase heart rate while being approached by a familiar human (Wascher *et al.,* 2011). We therefore need to consider the possibility that the observed changes in heart rate might not be representative of what would be observed in unhabituated wild animals, who might display even more pronounced responses or avoid areas in which fireworks occur altogether.

The physiological response we presently describe is likely associated with increased activity and a behavioural response. Anecdotally greylag geese from the studied population have been observed to take flight during the fireworks and circle over the lake Almsee, which is the roosting site (C.A.F. Wascher, personal observation). Hence, we suggest that the increase in heart rate in response to fireworks is likely to be caused by both, increased physical activity and psychological stress (Wascher, 2021). Wild birds have been shown to take flight for 45 minutes during New Year’s Eve fireworks (Shamoun-Baranes *et al.,* 2011); our results suggest an even longer response than this, heart rate only returned to baseline levels between 0200 and 0300, indicating that geese respond to the fireworks for 2 hours, which may also be due to the fact that firework activity does not sharply end a few minutes after midnight, but may occur as single crackers or rockets 1–2 hours into the new year. As we did not conduct behavioural observations, we cannot tell if geese took flight, for how long the geese were in the air and if all focal individuals did so; however, in different contexts, heart rate has been described to return to baseline levels within seconds after a stressor (Wascher *et al.,* 2008b; Wascher *et al.,* 2011; Wascher, 2021).

We have previously, shown that heart rate and body temperature vary—indicative of energy expenditure—profoundly across annual and daily cycles, generally decreasing during winter as compared with summer and significantly increasing during the reproductive period (Wascher *et al.,* 2018). Average daily heart rates in this previous study varied on average 22% between summer and winter, whereas body temperature was about 1° lower in winter compared with summer values. Here, we show heart rate increasing by 96% and body temperature increasing by 3% (about 1°) in the first hour of the new year, suggesting that New Year’s Eve celebration present a major stressor affecting individuals energy expenditure. Further, social contexts have been shown to be strong modulators of heart rate (Wascher *et al.,* 2008b; Wascher *et al.,* 2014) and of course, heart rate increases during locomotion and evidently stressful situations (i.e. a dog on the leash, the geese were not habituated to; Wascher *et al.,* 2011; Wascher and Kotrschal, 2013). Disturbance in response to fireworks is not only very likely stressful, but also presents an energetic cost to the geese (Butler and Woakes, 1980; Wascher *et al.,* 2018; Halsey *et al.,* 2019) and causes a disruption of their night rest period (Raap *et al.,* 2017; Aulsebrook *et al.,* 2020; Grunst *et al.,* 2021); in addition, birds are at risk of becoming disoriented (Van Doren *et al.,* 2017).

Although there were pronounced individual differences in heart rate and body temperature responses, these were not significantly affected by sex and age. Differences in heart rate between the sexes have previously been described as depending on season and only apparent during the reproductive season (Wascher *et al.,* 2018). Outside the reproductive season, differences between the sexes are context dependent: for example, male individuals having a higher heart rate during agonistic encounters compared with females (Wascher *et al.,* 2012). We did not describe an effect of age on the physiological response, showing no indication of the geese to either habituate or sensitize to the fireworks over time (Riemer, 2020). Both predictability and unpredictability as well as personality have been shown to affect behavioural and physiological stress response and depending on context can increase or decrease the response to stimuli (Bassett and Buchanan-Smith, 2007). However, it is questionable whether wild animals perceive New Year’s Eve celebrations as predictable events, as they only occur once a year.

To conclude, our results show that New Year’s Eve fireworks cause a substantial physiological response in greylag geese. A better understanding of the effects of anthropogenic disturbance onto wildlife can be useful for wildlife conservation attempts, and our study is one of few showing negative effects of fireworks onto wildlife. A clear recommendation from our and other studies is to avoid fireworks in nature areas altogether.

## Declarations

### Ethical Approval

This study was approved under an animal experiment license issued by the Austrian Ministry of Science GZ68.210/41-BrGT/2003. Before and after completion of the study, geese remained in the non-migratory, free-roaming flock of greylag geese in the Almtal.

### Consent for publication

Not applicable.

## Funding

This work has been funded by Austrian Science Fund (P15766-B03 and P18744-B03 to K.K.). The funder had no role in study design, data collection and analysis, decision to publish or preparation of the manuscript.

## Competing interests

The authors declare that they have no competing interests.

## Author Contributions

C.A.F.W. conceptualized the study, curated and analysed the data. C.A.F.W., W.A. and K.K. wrote the original draft of the paper and contributed to reviewing and editing. All authors read and approved the final manuscript.

## Availability of data and materials

The data and R code are provided via the platform Zenodo (10.5281/zenodo.6752634). https://zenodo.org/record/6752634#.YrhJvBPMLeo
